# Effect of probiotics on the meat flavour and gut microbiota of chicken

**DOI:** 10.1038/s41598-017-06677-z

**Published:** 2017-07-25

**Authors:** Yan Wang, Jing Sun, Hang Zhong, Nianzhen Li, Hengyong Xu, Qing Zhu, Yiping Liu

**Affiliations:** 1 Farm Animal Genetic Resources Exploration and Innovation Key Laboratory of Sichuan Province, Sichuan Agricultural University, Chengdu Campus, Chengdu, 611130 China; 2 Key laboratory of pig industry science, ministry of agriculture, Chongqing Key laboratory of pig industry science, Chongqing Academy of Animal Science, No. 51 Changlong Avenue Rongchang County, Chongqing, 402460 China

## Abstract

To date, no report has demonstrated the use of beneficial microbes for contributing to the flavour characteristics and gut microbiota diversity of chicken. Here, we selected six probiotics obtained from our laboratory and supplemented them in six different combinations to 420 newborn male Qingjiaoma chickens under the same controlled living environment (60 birds, no probiotic supplements). The results showed that chicken supplemented with *Bacillus* species showed beneficial effects in body weight. Acetate is the major fermentation production in the chicken caecum, and chicken supplemented with *Pediococcus pentosaceus* had the average higher short chain fatty acids (SCFAs) contents. In chicken caecal microflora, the abundance of *Bacteroidetes* bacteria was positively correlated with the content of propionate, butyrate, and isobutyrate, whereas an increase in acetate content was positively correlated to the abundance of *Firmicutes*. Compared to chickens without probiotic supplement, chickens supplemented with *P*. *pentosaceus* had more characteristic flavour compounds in the sampled breast meat, especially higher concentrations of (E)-2-heptenal, (E,E)-2,4-nonadienal, and certain C6-C9 unsaturated fatty acids. This resulted in a stronger chicken-fatty or fatty odour which directly improved the flavour. These findings suggest that probiotics can improve chicken meat flavour and increase gut microbiota diversity.

## Introduction

It is generally known that the diverse gut microbiota play an important role in host metabolism, nutrient digestion, growth performance and health of the host^1–4^. However, during the livestock production process, the widespread use of antibiotics and other drugs not only changs the gut micro-ecosystem but also causes the emergence of pathogenic bacteria resistant to antimicrobials, which has seriously threatened animal husbandry and human health^5, 6^. Therefore, the search for green and pollution-free additives to improve the immunity and nutrient utilization of livestock and poultry is an inevitable trend in green animal husbandry development in the 21^st^ century.

Probiotics are new green additives developed in recent years and are defined as mono-or mixed cultures of living microorganisms that beneficially affect the host animal by modulating gut microbiota in livestock^7–9^. Meanwhile, probiotics have been shown to reduce disease risk, possibly through a reduction in the proliferation of pathogenic species, maintaining microbiota balance in the gut and increasing resistance to infection^10^. For example, Pascual *et al*.^11^ reported that probiotics have a preventive effect against *Salmonella* ^11^. In addition, probiotic application has been reported in the poultry industry with an emphasis on their influence on the growth performance of chickens and their carcass compositions^12, 13^. In this context, recent studies reveal that probiotics supplements in chicken also improve pH, colour, water-holding capacity, fatty acid profile and oxidative stability in fresh meat^14, 15^. However, most research in this field has focused on how probiotics modulate the growth performance and microbial composition in livestock and poultry. Very few studies have comprehensively investigated the effects of probiotics on the diversity of the gut microbiota. In our previous study, we found that free-range chickens had abundant flavour compounds, high levels of ethyl hexanoate and beta-ocimene, and distinct bacterial strains in the caecal microbiota compared to those in caged chickens. Therefore, we hypothesized that probiotics isolated from the intestines of free-range chickens can improve meat flavour and promote animal health by modulating gut microbiota.

Thus, in the present study, we evaluated the effect of six different probiotics on the flavour characteristics and gut microbiota diversity in chickens using multiple approaches, including the 16S rRNA gene metagenome and HS-SPME analysis. The specific research aims were (1) to characterize the flavour compounds of chicken in different probiotic groups; (2) to characterize the influence of different probiotic supplements on enzyme activities in the digestive tract and bacterial fermentation in chicken caeca; (3) to characterize and compare the bacterial diversity of chicken caecal microbiota in different probiotic supplements groups; (4) to clarify the association among gut microbiota, caecal fermentation products and digestive enzyme activities and (5) to explore whether these correlations will influence meat quality, flavour compounds and gut microbiota composition of chickens.

## Materials and Methods

### Ethic statement

All animal work was conducted according to the guidelines for the care and use of experimental animals established by the Ministry of Science and Technology of the Peoples’ Republic of China and was approved by the Committee on Experimental Animal Management of the Sichuan Agricultural University, permit number 2014–18.

### Bacterial strain selection and tolerance measurement

A total of six strains, including *Pediococcus pentosaceus*, *Bacillus cereus var*. *albolactis*, *Bacillus macerans*, *Bacillus subtilis*, *Lactobacillus plantarum* and *Issatchenkia orientalis* were used as candidate probiotic strains for all experiments in this study. These strains were isolated from the mucous membrane of various intestinal segments (jejunum, ileum and caecum) of 154-day-old Caoke chickens (n = 6) and Qingjiama chickens (n = 6) that raised in a free-range system. Culture media for the experimental strains used de Man, Rogosa, and Sharpe (MRS) medium for *Lactobacillu*s *spp*, *Pediococcus* and yeast, and a common nutrition broth (CNB) medium was used for *bacillus spp* (shown in Table S1). To test the acid tolerance levels of the strains, simulated gastric juice was prepared according to Appendix XII A of the Pharmacopoeia of the People’s Republic of China (2010 Edition) set by the drug regulatory agency of the State Council (http://www.chp.org.cn). Experimental strains were injected into sterilized broth media (50 ml) and cultivated at 37 °C for 18 hours. Each strain suspension (0.1 ml) was inoculated into tubes with 10 ml of simulated gastric juice containing 0.1 g pepsin at a specific pH (pH2, pH3 and pH4), which were adjusted using dilute HCl. *Lactobacillu*s *spp* and *Pediococcus* were incubated at 37 °C and yeast at 30 °C for 4 h. Serial decimal dilutions in MRS agar medium for *Lactobacillu*s *spp*, *Pediococcus* and yeast and CNB agar medium for *bacillus spp* were prepared, after which aliquots (1 ml) of the dilutions were spotted onto MRS or CNB agar plates to determine the number of surviving cells. Each determination was performed in duplicate. Bile tolerance was measured according to the method of Park with slight alterations (1999)^16^. Briefly, 0.1 ml of strain suspension was inoculated into tubes with 10 ml bile salt solution, which contained 0.05 g NaCl, 0.01 g trypsin powder and varying concentrations of chicken bile salt (0.1%, 0.2% and 0.3%, Sigma Aldrich). An agar plate without bile salt was used as a control. To count cells displaying bile acid tolerance, the same methods as those used to measure acid tolerance were performed, as mentioned above.

### Determination of the degree of hydrolysis of strains

Another important characteristic of a microbial strain employed for probiotic use is the degree of hydrolysis (DH) in the fermented feedstuffs given to chickens. Protein hydrolysis in feedstuff is mainly aimed at producing small polypeptides protein moieties. This physical method can increase the nutritional value of insoluble protein sources and improve the organoleptic and functional value of a variety of foodstuffs^17^. To determine the degree of protein hydrolysis in fermented feedstuffs by each specific strain, the Formol-titration method was employed (2001)^18^. We determined the formol number that responds to amino nitrogen content in microbially fermented feed by acidimetric titration (GB/T 12143.2-89; http://www.chinagb.org/).

### Production of probiotic powders

Strains selected according to their acid and bile tolerance levels were used to prepare the probiotic powder. Corn, soybean meal and wheat bran, which are typically used in industry, were used after autoclave sterilization as fermented solid media. The probiotic powder was produced as follows: corn, soybean meal and wheat bran mixed at a ratio of approximately 12:6:1 were sifted using an officinal disintegrator (Shanghai PuHeng industrial Ltd. Co., Shanghai, China) and used as fermentation substrates. The fermentation conditions of each strain were selected for their respective optimal conditions (Table S1). After continuous 72 h of solid-state fermentation, the fermentation product was combined with an equal weight of sifted corn flour (sieve size 0.422 ± 0.04 mm) dried under natural conditions and shattered into powder (sieve size 0.422 ± 0.04 mm) to allow the detection of a viable count per fermentation product. In total, there were six types of bacterial powder products produced for use in the present study. Each powder product was packaged into 200-gram quantities in light-tight envelopes and vacuum sealed until use.

### Animal experiments

Four hundred twenty 1-day-old commercial male Qingjiaoma (QJM) A-strain chickens were provided by the Meishan Wens Poultry Breeding Ltd. Company (Sichuan, China) and randomly divided into six trial groups (i.e., Group1 to Group6) and the control group (Group7). Each group contains 60 birds and randomly consists of two repeating groups (30 birds per repetition). Chickens were grouped into seven pens (1250 × 70 × 30 cm) in the experimental poultry house of Sichuan Agricultural University and transferred into single cages (36 × 55 × 40 cm) at 39 days old. All birds were given free access to diet and clear water and were vaccinated according to the routine vaccination programme provided by the Wens Company (Table S2).

From day 1 to day 42 (the brooding period), 0.2% *Bacillus*-, *Lactobacillus*- and *Pediococcus*-based probiotic fermented products were added to the diet of all chickens, and from day 43 to day 76 (the growing period), 0.15% probiotic fermented products were added, whereas *Pichiaceae*-based probiotic fermented product was provided to the diet in 0.25% of feed at the brooding phase and 0.15% of feed during the growing period. The use of probiotic products was grouped principally by the host origin of strains and the degree of hydrolysis (Table S2). For example, Group1: *P*. *pentosaceus* (6.86 × 10^9^ CFU/g) + *B*. *subtilis* (1.60 × 10^9^ CFU/g,); Group2: *P*. *pentosaceus* + *Bacillus cereus var*. *albolactis* (5.4 × 10^8^ CFU/g); Group3: *I*. *orientalis* (1.6 × 10^8^ CFU/g) + *B*. *macerans* (6.9 × 10^8^ CFU/g,); Group4: *B*. *cereus var*. *albolactis* + *I*. *orientalis* + *L*. *plantarum* (8.35 × 108 CFU/g), which had a similar degree of protein hydrolysis to feedstuff. Group5: *B*. *macernas* (6.9 × 10^8^ CFU/g) was supplied to chickens in Group 5 alone; to observe the influence of *P*. *pentosaceus* alone that had the highest degree of protein hydrolysis (DH = 63.41%), it was used alone to feed Group 6 chickens. No probiotics supplement was included in the diet of the Group 7 chickens, as they served as the control group. The process of probiotic supplementation to the diets of chickens lasted from 1 to 76 days of age.

### Growth monitoring and sample collection

The mortality and elimination rate of birds were recorded daily, and the body weight and feed consumption for each group were surveyed once per week. We monitored the growth of all chickens throughout their life-cycle until 76 days of age, at which point we randomly selected six birds per group (three birds per repetition) to sacrifice by exsanguination. Growth performance and slaughter traits including the carcass weight, breast meat percentage, thigh muscle percentage, abdominal fat percentage and subcutaneous fat thickness were measured and compared.

The gut contents in different intestinal positions including jejunum, ileum, caecum and duodenum were then collected. Considering the huge dissimilarity in gut bacterial composition between individuals of a similar group^19^, six intestinal samples and six intestinal contents from each group were pooled and transferred to 5 ml tubes with 500 μl RNAlater^®^ solution (Ambion, Austin, USA). All mixed samples were used for bacterial 16S rDNA gene V3 region sequencing (BGI, Shenzhen, China).

### Analysis of volatile constituents

To determine the meat flavour compositions of the different treatment chickens, approximately 10 grams of tissue from each breast sample per group were collected and minced using a meat grinder. To guarantee precise and reliable results, all experiments in the present study were performed in triplicate.

Headspace-solid phase microextraction (HS-SPME) analysis: A 75 μm Carboxen^TM^/Polydimethysiloxane (CAR/PDMS) StableFlex fibre (Supelco, Bellefonte, PA, USA) was exposed to each sample and placed in a 15 ml vial for 35 min at 70 °C; the fibre was then injected into the gas chromatograph injection unit.

Analysis of GC-MS: quantitative analyses of the meat volatile compounds was performed using a GC-MS 2010 Series system with a 57318 SPME manual holder equipped with a DB-5MS (30 m × 0.25 mm I.D., 0.25-μm film thickness) (Shimadzu, Tokyo, Japan) fused-silica capillary column coupled to an Agilent model 5977N MSD mass spectrometer (MS) (Agilent model 5977N MSD mass spectrometer). The GC conditions in the GC-MS analysis were as follows: the oven temperature was held at 40 °C for 5 min, followed by an increase to 60 °C at 5 °C/min for 1 min, to 130 °C at 6 °C/min for 1 min, and increased from 130 °C to 230 °C at 10 °C/min and held for 3 min. The injector temperature was maintained at 250 °C. The volatile compounds in the extracts were identified by comparison of the retention indices and MS fragmentation patterns to published data (obtained from NIST 05, NIST 08, PESTEI 3, and PESTNCI 3 mass spectral libraries).

Finally, we used SPSS software version 19.0 to perform one-way ANOVA analysis to compare the significant difference of the concentrations of flavour compounds observed between different treatment groups and the control group.

### Analysis of SCFAs in chicken caecal samples

Caecal contents of each individual bird from all seven groups were divided into two parts: one was used to determine the concentration of four short chain fatty acids (SCFAs) including acetate, propionate, butyrate and isobutyrate, and the other was blended into an integrated sample for analysis of gut microbiota. In addition, 500 mg of frozen caecal samples were used to determine SCFAs. Briefly, caecal samples were homogenized after adding 3 ml of ultrapure water and centrifuged at 10,000 × g for 10 min at 4 °C. One milliliter of supernatant was homogenized with 0.2 ml 25% metaphosphoric acid and placed on ice for at least 30 min, and were then centrifuged at 10,000 × g for 10 min at 4 °C. The concentrations of SCFAs were determined in a 1:25 dilution of 500 μL of supernatant. Four calibration curves were prepared to quantify the four SCFAs using the Multiple Point External Standard method. Gas chromatograph (Mead) determination was employed to quantify SCFA content using a Shimadzu GC-2010ATF instrument. The chromatographic column specifications were WAX-DA 30 m × 0.25 mm, 0.5-μm film thickness. Using a splitless inlet, the injection volume and injector temperature were 1 μL and 220 °C, respectively; the GC oven temperature programme was as follows: initial temperature 90 °C for 0.50 min, then ramped to 150 °C at 5 °C/min and lasted 7 min; the temperature of the FID detector was at 230 °C; the make-up flow, hydrogen flow and air flow were 30 ml/min, 40 ml/min and 400 ml/min, respectively. The SCFAs concentrations in caecal samples were expressed in millimoles per gram (mmol/g) of caecal content. We used SPSS software version 19.0 to perform ANOVA analysis for determining the statistical significance of differences observed between treatment groups and the control group.

### Determination of digestive enzymes of chicken intestinal tract

The activities of trypsin, lipase, and amylase in the intestinal contents of the four intestinal segments (duodenum, jejunum, ileum, and a pair of ceca) were measured at 76 days of age. In brief, atleast 1 ml of caecal content sample from each chicken was diluted with 5 ml of 0.85% saline water and homogenized for 5 min. The suspension was centrifuged (12000 × g for 20 min at 4 °C) to obtain a supernatant of 20% intestinal juices and subpackaged into 1.5-mL sterile tubes. When the activities of digestive enzymes in different intestinal sections were detected, the intestinal contents of three individuals from the same test group were mixed to create an integrated sample for all seven groups. In total, 56 integrated samples were prepared, and the determination methods were performed according to the National standard of the People’s Republic of China (GB/T 23535–2009 for lipase, GB 8275–2009 for amylase, http://www.chinagb.org/). The bicinchoninic acid (BCA) assay for total protein concentration in gut contents^20^ and the activity of trypsin was measured by the increase in absorbance (A) at 263 nm^21^. Each sample in the intestinal tissue was measured three times to obtain stable, reliable results. An 8200 UV/VS Spectrophotometer (JASCO, Tokyo, Japan) was used for the quantitative analysis of enzyme activities. We used SPSS software version 19.0 to perform the General Lineal Model for multivariable analysis to determine the statistically significant differences of three digestive enzymes observed between treatment groups and the control group in four different intestinal sections.

### DNA extraction, PCR amplification, and high-throughput sequencing

To analyse the composition of QJM chicken intestinal microbiota in different groups, the microbial DNA of the caecal was extracted using the QIAamp-DNA Stool Mini Kit (Qiagen, Hilden, Germany) according to the manufacturer’s protocol. The concentration and quality of the extracted genomic DNA were assessed using a Quant-IT^TM^ dsDNA BR Assay Kit (Invitrogen) and NanoVue Plus^TM^ spectrophotometer (Thermo Scientific, Wilmington, DE, USA). The integrity of the extracted DNA was determined using electrophoresis on a 1% agarose gel. The DNA extracts were used as templates to amplify the V3 hypervariable region of the 16S rDNA gene with the primers 338 F (5′-ACTCCTACGGGAGGCAGC-3′) and 533R (5′-TTACCGCGCCTGCTGGCAC-3′)^22^. Amplification was performed in a total volume of 20 μl, and three replicates were performed for each sample. Each replicate consisted of 1 μL (100 ng) of DNA template, 1 μL HotStarTaq^®^ Plus Master Mix Kit (contains HotStarTaq plus DNA polymerase, PCR buffer with 3 mM MgCl_2_, and 400 μM of each dNTP) (QIAGEN, Shanghai, China), 0.8 μL of each 5 μM primer and double deionized water. After 3 min of denaturation at 95 °C, 30 cycles of 30 s at 95 °C, 30 s at 55 °C, and 45 s at 72 °C were performed, followed by a final extension for 10 min at 72 °C. Three replicates of each sample were pooled and purified using an AxyPrep DNA gel extraction kit (Axygen Bioscience, Union City, USA) and quantified using a Qubit dsDNA BR Assay Kit (Invitrogen, Shanghai, China). The purified amplicons were sequenced on an Illumina HiSeq TM 2000 platform by BGI Co., Ltd. (Shenzhen, China).

### Bioinformatic and statistic analysis

Overlap Assembler Software was used for 16S rDNA gene PCR amplicons, which had a minimum length of 30 bp. No mismatches and N bases were allowed in the overlapping area. The primer sequences were removed from each obtained sequence and short reads (<55 bp) were eliminated for further tag analysis. Mothur 1.11.0 was used to reduce redundancy and to screen unique tags^23^; taxonomic assignment was determined using BLASTN, and a maximum e-score of 1e-05 was used as an approximation of the taxonomic determination of a given sequence.

The richness and biodiversity of the caecal samples were determined by Alpha diversity. We used the Chao 1 and ACE indices to estimate the richness related to the number of observed Operation Taxonomic Units (OTUs). The Shannon and nonparametric Shannon indices were used to estimate the biodiversity of the OTUs at a cut-off of 0.03-distance unit. A ward method in the pvclust package in R (V.2.9.1) was used to study the multivariate clustering of ceacal samples (http://www.is.titech.ac.jp/~shimo/prog/pvclust/). Principal components analysis (PCA) of overall diversity of microbial communities based on family level was performed using Bray-Curtis distance to compare all samples. The difference of the performance, carcass traits, flavour component, SCFA and enzyme content between probiotic-treated group and control group were analysed with the Independent-Samples T procedure of SPSS. We estimated the correlations between phylum abundance and SCFAs in the chicken intestinal tract using Canonical Correlation analysis (PROC CANCORR program in CCA Package) in SAS 8.1. All results were considered statistically significant at P < 0.05 among different groups.

## Results

### Comparison of performance and carcass traits through the addition of probiotics

Data on performance indices are summarized in Table 1. The body weight of chickens was checked every seven or eight days. The results showed that the probiotics had a growth promoting effect on the average body weight of chickens (Table 1 and Supplementary Fig. S1). The average body weight from probiotic Group 3 was significantly higher than that from the control group (i.e., Group 7, P = 0.0055). Although the other probiotic groups, including Group 1, Group 2, Group 4, Group5 and Group 6 all showed positive effects on average body weight compared to the control group, the differences were not significant (P > 0.05) (Table 1). Meanwhile, we found that the feed conversion ratio (FCR) of Group 1 (2.29) was reduced compared to the control group, followed by Groups 3 and 5 (FCR = 2.36 and FCR = 2.37, respectively), but there were no significant differences (P > 0.05) (Table 1). Therefore, considering the feed conversion ratio, the probiotic group is superior to the control group.

**Table 1:** Growth and meat production at 74 days of age of Qingjiaoma chickens given different probiotic-supplemented fermented feed.

Performance Metric	Chickens fed with a variety of probiotic supplements						Control Group
	Group 1	Group 2	Group 3	Group 4	Group 5	Group 6	Group 7^a^
Average body weight (g)	3132.67 ± 211.5	3140.00 ± 222.3	3201.33 ± 290.6^*^	3013.08 ± 292.4	3060.00 ± 271.1	3086.67 ± 250.5	2928.67 ± 256.5
Carcass percentage (%)	80.15 ± 0.028	79.34 ± 0.027	80.61 ± 0.022	80.13 ± 0.028	79.29 ± 0.014	80.53 ± 0.025	80.19 ± 0.035
Breast muscle percentage (%)	35.98 ± 0.027	37.80 ± 0.028	35.72 ± 0.033	34.95 ± 0.043	33.25 ± 0.046	36.15 ± 0.027	35.64 ± 0.38
Thigh muscle percentage (%)	37.13 ± 0.029	38.40 ± 0.025	37.63 ± 0.017	38.80 ± 0.034	35.43 ± 0.079	43.50 ± 0.110	35.00 ± 0.105
Abdominal fat percentage (%)	2.16 ± 0.008	1.98 ± 0.007	1.73 ± 0.008	2.14 ± 0.007	2.61% ± 0.011	2.00 ± 0.009	1.46 ± 0.009
Subcutaneous fat thickness (cm)	0.68 ± 0.08	0.76 ± 0.13	0.55 ± 0.05	0.61 ± 0.21	0.66 ± 0.12	0.73 ± 0.086	0.64 ± 0.28
Feed conversion ratio	2.29	2.45	2.36	2.53	2.37	2.46	2.55
Morbidity and mortality cases	2^b^	2^c^	0	1^d^	0	0	3^e^

Asterisk (*) represents the significant difference of average body weight of Qingjiaoma chickens between Group 3 and the control group Group 7 (*P value* = 0.0055).

^a^Group 7 is the negative control group of chickens were fed without any probiotic supplement.

^b^One bird in Group 1 was crushed by misplaced feed utensils at Day 1.

^c^One bird in Group 2 fell from the cage and hurt its leg at 5 days of age. To control the experimental conditions, this bird was eliminated from our analysis.

^d^One bird was eliminated from this group due to a wry neck caused by the improper subcutaneous injection of Bird Flu vaccine, to control the experimental condition this bird was eliminated for our analysis.

^e^Two birds in the control group were eliminated by debeaking stress, which induced a very weak capability of food intake beginning at day 8, so we eliminated them at Day 11, respectively; At Day 17, another bird was eliminated with a wry neck that caused by the improper subcutaneous injection of Bird Flu vaccine.

In addition, Table 1 shows relative weight means of muscle as a percentage of slaughter weight, breast muscle weight, thigh muscle weight, abdominal fat weight, and subcutaneous fat thickness. There were no significant differences in carcass traits of chickens in probiotics groups and control groups (P > 0.05). Among them, slaughter percentage, breast meat percentage, thigh muscle percentage and abdominal fat percentage were slightly increased with the Group 3 supplement compared to the control group but the difference was not significant (P > 0.05). Chickens supplemented with probiotics in Group 4 and Group 5 had lower breast muscle percentages (34.95% and 33.25%) and higher thigh muscle percentages (38.80% and 35.43%) compared to the control group (35.64%, 35.00%); the values did not differ significantly between these groups (P > 0.05). In addition, compared to the control group, Group 3 and Group 4 probiotic supplementation was shown to reduce the subcutaneous fat thickness (P = 0.087 and P = 0.057, respectively).

### Improvement in meat flavour component through the addition of probiotics

To evaluate the flavour substances of chicken, the breast muscle was analysed using a GC/MS technique. The results for the partial main volatile compounds are presented in Table 2. In total, 43 compounds were detected in Group 7 without probiotic supplementation, and 52, 60, 54, 54, 49 and 55 compounds were detected in Group 1, Group 2, Group 3, Group 4, Group 5 and Group 6, respectively.

**Table 2:** Comparison of partial flavor components in breast meat of birds with and without probiotic additive.

Component	RT^a^	Average concentration (μg/g)						Control group
		Chickens fed with a variety of probiotic supplements						
		Group 1	Group 2	Group 3	Group 4	Group 5	Group 6	Group 7
2-Butanone, 3-methyl-	2.700	1.15e-04 ± 5.90e-05	3.73e-05 ± 5.56e-06	3.02e-06 ± 3.36e-07 (0.025)	1.31e-06 ± 2.50e-07 (0.023)	1.73e-06 ± 6.10e-07 (0.023)	1.23e-05 ± 2.02e-06 (0.029)	1.1.81e-04 ± 4.32e-06
Butanal, 3-methyl	3.492	6.74e-05 ± 5.45e-05	4.85e-06 ± 1.01e-06 (0.026)	5.99e-05 ± 3.92e-05	1.21e-05 ± 3.03e-06 (0.026)	1.06e-05 ± 5.61e-06 (0.016)	3.97e-06 ± 2.02e-06 (0.020)	7.72e-05 ± 5.14e-05
Pentanal	4.133	2.01e-04 ± 8.05e-05	1.53e-04 ± 1.27e-05	1.96e-04 ± 5.18e-05	1.46e-04 ± 4.87e-05	1.74e-04 ± 4.67e-05	1.51e-04 ± 3.96e-05	1.71e-04 ± 8.16e-05
2-Butanone, 3-hydroxy-	5.037	9.76e-05 ± 6.48e-05	3.82e-06 ± 1.29e-06	3.02e-06 ± 1.15e-06	2.24e-06 ± 1.34e-06	1.29e-06 ± 9.15e-07	—	1.81e-04 ± 1.55e-04
(E)-2-Pentenal	5.758	—	—	—	—	1.08e-06 ± 9.15e-07	—	—
1-Pentanol	6.167	7.74e-05 ± 4.46e-05	6.75e-05 ± 4.91e-05	1.29e-04 ± 9.11e-05	6.15e-05 ± 2.43e-05	5.51e-05 ± 1.18e-05	7.37e-05 ± 2.69e-05	2.00e-05 ± 7.83e-06
(E)-2-Decenal	6.750	—	—	—	—	—	4.09e-06 ± 2.36e-06	—
Hexanal	7.256	8.91e-04 ± 2.68e-04	1.12e-03 ± 3.48e-05	1.06e-03 ± 4.80e-04	8.29e-04 ± 1.86e-04	1.29e-03 ± 1.96e-04	1.34e-03 ± 1.08e-04	1.23e-03 ± 3.14e-04
(E)-2-hexenal	9.058	1.28e-05 ± 7.41e-06	2.06e-05 ± 7.37e-06	5.60e-06 ± 3.97e-06	1.17e-05 ± 8.27e-06	1.73e-05 ± 2.44e-06	2.1e-05 ± 3.80e-06	3.32e-06 ± 1.73e-06
1-Hexanol	9.663	1.96e-05 ± 1.12e-05	2.93e-05 ± 1.10e-05	1.64e-05 ± 1.39e-06	1.12e-05 ± 5.26e-06	4.51e-05 ± 1.73e-05	2.63e-05 ± 4.51e-06	8.15e-06 ± 5.40e-06
Heptanal	10.735	1.07e-04 ± 2.85e-05	2.16e-04 ± 2.59e-05 (0.046)	9.21e-05 ± 5.99e-05	9.09e-05 ± 3.66e-05	2.24e-04 ± 8.78e-05 (0.031)	1.55e-04 ± 3.23e-05	1.27e-04 ± 4.53e-05
Styrene	10.308	5.89e-05 ± 3.227e-05	1.04e-04 ± 3.51e-05	6.38e-05 ± 1.99e-05	3.06e-05 ± 7.65e-06	5.29e-05 ± 1.98e-05	5.92e-05 ± 2.98e-05	4.18e-05 ± 9.90e-06
Oxime-, methoxy-phenyl-	10.994	6.19e-05 ± 1.77e-05	4.72e-05 ± 2.03e-05	1.33e-04 ± 5.53e-05 (0.013)	1.28e-04 ± 4.24e-05 (0.018)	1.02e-04 ± 2.44e-06	9.88e-05 ± 2.35e-05	4.06e-05 ± 2.24e-05
Butyrolactone	11.192	7.96e-06 ± 3.23e-06	2.67e-06 ± 5.40e-07	6.89e-06 ± 2.42e-06	5.20e-06 ± 2.50e-07	8.20e-06 ± 7.93e-07	2.05e-06 ± 3.54e-07	5.47e-06 ± 8.20e-07
(E)-2-heptenal	12.541	3.44e-05 ± 2.26e-05	1.51e-04 ± 1.67e-05 (0.0020)	4.35e-05 ± 1.73e-05	5.97e-05 ± 8.27e-06	8.73e-05 ± 3.17e-05	1.64e-04 ± 1.43e-05 (0.0010)	9.47e-05 ± 1.89e-05
Benzaldehyde	12.708	4.04e-05 ± 1.23e-05	7.22e-05 ± 4.16e-05	6.89e-05 ± 7.78e-06	3.70e-05 ± 9.77e-06	7.18e-05 ± 2.88e-05	—	5.46e–5 ± 7.14e-06
Dimethyl trisulfide	12.908	4.99e-06 ± 2.88e-06	—	1.03e-05 ± 7.31e-06	—	—	—	—
1-Heptanol	13.000	3.21e-05 ± 1.45e-05	7.09e-05 ± 1.35e-05	3.06e-05 ± 4.26e-06	2.93e-05 ± 8.99e-06	7.81e-05 ± 1.50e-05	5.69e-05 ± 2.73e-06	2.67e-05 ± 4.32e-06
Octanal	13.975	1.23e-04 ± 3.05e-05	2.14e-04 ± 7.29e-05 (0.038)	1.56e-04 ± 2.85e-05	1.25e-04 ± 5.03e-05	2.62e-04 ± 1.51e-05	2.13e-04 ± 3.67e-05	1.35e-04 ± 3.38e-05
1-Octen-3-one	13.192	4.46e-06 ± 3.27e-06	6.11e-06 ± 5.40e-07	7.54e-06 ± 1.52e-06	3.90e-06 ± 2.50e-06	7.77e-06 ± 5.49e-06	8.32e-06 ± 6.25e-07	2.56e-06 ± 4.41e-07
1-Octen-3-ol	13.316	—	1.20e-04 ± 1.49e-05	1.22e-04 ± 8.65e-05	1.42e-06 ± 1.00e-06	1.28e-04 ± 2.32e-05	1.13e-04 ± 7.58e-06	2.29e-05 ± 3.96e-06
(E,E)-2,4-Heptadienal	14.250	1.50e-05 ± 1.05e-05	1.91e-05 ± 1.35e-05	4.31e-07 ± 3.04e-07	—	—	1.91e-05 ± 1.00e-06	—
2-Acetylthiazole	14.500	1.14e-05 ± 5.66e-07	2.67e-06 ± 6.64e-07	1.29e-06 ± 6.07e-07	—	2.16e-06 ± 1.97e-06	6.55e-06 ± 4.96e-07	3.44e-06 ± 4.86e-07
D-limonene	14.749	2.20e-05 ± 1.06e-05	8.42e-05 ± 7.56e-06 (0.005)	3.32e-05 ± 1.73e-05	2.89e-05 ± 8.62e-06	5.87e-05 ± 1.07e-05	7.18e-05 ± 1.44e-05 (0.019)	2.74e-05 ± 5.13e-06
4-Ethylcyclohexanol	15.008	2.5e-06 ± 1.76e-06	3.30e-05 ± 4.014–06	1.28e-05 ± 9.08e-06	2.32e-05 ± 2.58e-06	1.78e-05 ± 5.81e-05	1.73e-05 ± 8.09e-06	8.66e-06 ± 3.24e-06
2,4-dimethyl-Cyclohexanol	15.305	1.53e-05 ± 2.27e-06	1.97e-05 ± 5.83e-06	8.18e-06 ± 4.50e-06	2.00e-05 ± 8.77e-06	1.93e-05 ± 2.75e-06	3.32e-05 ± 1.38e-05 (0.0015)	8.67e-06 ± 3.24e-06
(E)-2-Octenal	15.600	3.78e-05 ± 1.83e-05	9.94e-05 ± 5.92e-06 (0.002)	4.24e-05 ± 1.57e-05	4.33e-05 ± 1.92e-05	8.96e-05 ± 3.27e-05 (0.0070	9.42e-05 ± 7.35e-06 (0.004)	4.02e-05 ± 2.22e-05
Benzeneacetaldehyde	15.242	4.64e-06 ± 1.00e-06	8.40e-06 ± 5.94e-06	3.45e-06 ± 2.43e-06	2.13e-06 ± 1.50e-06	—	—	—
1-Octanol, 2-butyl-	15.733	2.02e-05 ± 4.50e-06	9.55e-07 ± 2.70e-07	2.15e-06 ± 1.52e-06	3.54e-07 ± 2.50e-07	1.73e-06 ± 1.22e-06	1.23e-06 ± 8.19e-07	—
Cyclohexanone, 4-(benzoyloxy)-	15.855	—	1.41e-05 ± 1.08e-06	—	1.10e-05 ± 7.77e-06	—	—	3.95e-06 ± 6.84e-07
(E)-2-Octen-1-ol	15.863	6.96e-06 ± 1.26e-06	8.40e-06 ± 9.26e-07	7.54e-06 ± 4.57e-06	—	—	1.19e-05 ± 1.42e-06	—
1-Octanol	15.969	4.53e-05 ± 8.83e-06	9.55e-05 ± 1.05e-05 (0.004)	4.78e-05 ± 3.38e-05	—	1.01e-04 ± 3.26e-05 (0.002)	8.02e-05 ± 8.42e-06 (0.022)	2.57e-05 ± 1.94e-05
Octadecanoic acid, ethenyl ester	16.619	3.00e-05 ± 2.52e-06	1.88e-05 ± 1.81e-06	3.05e-05 ± 1.64e-05	2.98e-05 ± 1.05e-05	1.97e-05 ± 1.74e-06	2.09e-05 ± 2.47e-06	—
Nonanal	16.886	1.49e-04 ± 2.54e-05	1.86e-04 ± 5.74e-05	1.39e-04 ± 8.13e-06	1.15e-04 ± 2.58e-05	2.16e-04 ± 1.10e-05 (0.050)	1.80e-04 ± 2.25e-05	1.46e-04 ± 2.94e-05
(E)-2-Nonen-1-ol	17.083	5.71e-06 ± 2.34e-06	2.93e-06 ± 2.25e-07	—	3.54e-07 ± 2.50e-07	—	1.19e-05 ± 1.42e-06 (0.045)	1.41e-06 ± 2.42e-06
3-Octadecyne	18.775	—	1.91e-05 ± 4.86e-06 (0.043)	8.62e-06 ± 2.12e-06	1.48e-05 ± 6.30e-06	1.89e-05 ± 6.53e-06	1.02e-05 ± 7.80e-06	1.29e-05 ± 2.71e-06
Cyclooctanemethanol	17.907	1.43e-06 ± 6.18e-07	1.27e-06 ± 6.62e-07	6.46e-07 ± 3.04e-07 (0.009)	1.31e-06 ± 5.41e-07	2.03e-06 ± 2.49e-07	1.36e-06 ± 8.52e-07	2.18e-06 ± 1.17e-06
Dodecanal	18.156	2.85e-06 ± 2.01e-06	—	—	—	—	1.77e-06 ± 1.44e-06	—
(E)-2-Nonenal	18.384	2.29e-05 ± 5.69e-06	3.18e-05 ± 1.10e-05	3.45e-05 ± 1.56e-05	2.28e-05 ± 5.87e-06	2.65e-05 ± 1.41e-05	3.07e-05 ± 5.53e-06	1.85e-05 ± 3.42e-06
2,6/3, 5-Dimethylbenzaldehyde	18.519	2.85e-06 ± 2.01e-06	5.86e-06 ± 1.7e-06 (0.046)	3.88e-06 ± 2.74e-06	4.25e-06 ± 3.00e-06	—	5.73e-06 ± 1.08e-06	1.41e-06 ± 1.03e-06
1-Nonanol	18.647	4.53e-05 ± 8.83e-06	2.67e-06 ± 8.10e-07	3.45e-06 ± 2.43e-06	2.13e-06 ± 1.50e-06	—	2.59e-06 ± 8.52e-07	—
2-n-Heptylfuran	19.183	—	3.06e-06 ± 2.10e-06	—	—	—	3.82e-06 ± 2.60e-06 (0.0180)	7.64e-07 ± 1.32e-07
Cis-4-decenal	19.252	—	1.91e-05 ± 1.08e-06	2.15e-06 ± 1.52e-06	—	3.02e-06 ± 2.13e-06	3.27e-06 ± 8.19e-07	—
Decanal	19.547	7.49e-06 ± 1.43e-06	8.02e-06 ± 2.50e-06	—	4.02e-06 ± 1.08e-06	9.63e-06 ± 3.06e-06 (0.048)	6.96e-06 ± 1.78e-06	4.97e-06 ± 2.13e-06
(E,E)-2,4-Nonadienal	19.838	1.78e-06 ± 1.26e-06	5.62e-06 ± 2.23e-06	2.58e-06 ± 0.00	3.90e-06 ± 2.75e-06	5.32e-06 ± 3.66e-06	5.05e-06 ± 1.70e-06	7.64e-07 ± 1.32e-07
1,3-Hexadiene,3-ethyl-2-methyl-	19.942	2.20e-05 ± 1.06e-05	6.88e-05 ± 7.56e-06	3.53e-05 ± 1.73e-05	2.84e-05 ± 2.21e-05	5.85e-05 ± 1.07e-05	7.18e-05 ± 1.44e-05	2.74e-05 ± 5.13e-06
2-nonenal, (E)-	20.936	2.29e-05 ± 5.69e-06	3.18e-05 ± 1.10e-05	3.45e-05 ± 1.57e-05	2.28e-05 ± 5.87e-06	2.65e-05 ± 1.41e-05	3.07e-05 ± 5.54e-06	1.85e-05 ± 3.42e-06
2-undecenal	23.133	1.96e-05 ± 0.00	8.33e-05 ± 8.91e-06	1.49e-05 ± 4.52e-06	2.52e-05 ± 4.76e-06	9.92e-05 ± 9.15e-07 (0.016)	2.35e-05 ± 1.70e-05	2.45e-05 ± 3.77e-06
Trans-3-Nonen-2-one	22.681	—	2.80e-06 ± 9.61e-07	—	1.42e-06 ± 1.00e-06	—	2.18e-06 ± 4.73e-07	—
2,5-Furandione, 3-dodecenyl-	23.948	—	—	—	5.32e-06 ± 2.00e-06	—	—	—
Trans-2-undecen-1-ol	23.965	1.78e-05 ± 8.91e-06	1.91e-05 ± 4.86e-06	8.62e-06 ± 1.21e-06	1.48e-05 ± 6.30e-06	1.89e-05 ± 6.53e-06	1.02e-05 ± 7.80e-06	2.18e-05 ± 1.57e-05
Eicosanoic acid	32.226	3.57e-07 ± 2.55e-07	1.80e-06 ± 8.82e-07 (0.0172)	—	2.48e-06 ± 1.75e-06	—	—	—

^a^RT means the retention time of each flavor component by HS-SPME-GC-MS analysis.

The dash (−) represents the specific flavor compound was not detected from the sampled breast meat in a certain group.

Figure in parentheses means the statistical significant difference *P* values of the concentration of certain flavor compound in chicken breast meat between certain treatment group and the control group Group 7.

Aldehydes and alcohols were the most important compounds identified in chickens using the GC/MS technique, followed by hydrocarbons, ketones and esters. Among them, (E,E)-2,4-nonadienal has low threshold values and a high impact on chicken meat aroma, its levels were higher in Groups 2, 5 and 6 compared to control Group7, but there was no significant difference (P > 0.05). Meanwhile, two C6-C9 unsaturated fatty aldehydes, including (E)-2-hexenal and (E)-2-nonenal have high concentrations in all probiotic supplementary groups compared to control Group 7, but the difference was not significant (P > 0.05). The concentration of (E)-2-heptenal and (E)-2-octenal in Group 2 and Group 6 showed a significant increase compared to control Group 7 (P = 0.002, 0.002, 0.001 and 0.004, respectively). Likewise, the flavour substances of caprylic aldehyde in Group 5 and Group 6 have significantly higher concentrations than in the control group (P = 0.002 and 0.039, respectively). In addition, for alcohols, we found that 1-octen-3-ol was detected in all groups except for Group 1, and its concentration in Group 2, Group 3, Group 5 and Group 6 was higher than in control Group 7; however, the difference was not significant (P > 0.05). The other important alcohols for meat quality is 1-Octanol, which has an oily, citrus, coconut note, green odour and fruity flavour. As shown in Table 2, the concentration of 1-Octanol in Group 2, Group 5 and Group 6 was significant higher than in control Group 7 (P = 0.004, 0.002 and 0.022, respectively).

In the present study, some flavour compounds were unique to certain groups, which can either directly or indirectly affect the flavour of chicken meat. Dimethyl trisulphide is a very important compound in meat with an overripe, asparagus, corn and vegetable-like flavour, but it was only detected in Groups 1 and 3. (E,E)-2,4-Heptadienal has a fat-like odour, which was not detected from the samples of control Group 7 but was detected in Groups 1, 2, 3 and 6. Trans-3-Nonen-2-one, which has stewed fruity odour with mushroom undertones, was also only found in Groups 2, 4 and 6. (E)-2-Decenal and Cis-4-decenal both have a chicken fatty odour, but were only found in Group 2 and Group 6. Therefore, Group2 and Group 6 contains more intense and more characteristically flavourful components.

### Comparison of SCFAs contents by caecal fermentation

The average concentrations (±SD) of caecal SCFAs in each group are shown in Table 3. Concentrations of acetic acid in Group 6 caecum were significantly higher than in control Group 7 (P = 0.015), but were significantly lower in Group1 and Group 2 than in control Group 7 (P = 0.026 and P = 0.034, respectively). Similarly, the concentration of isobutyric acid and butyric acid in Group 1 and Group 6 was significantly higher than those in control Group 7, respectively (P = 0.044 and P = 0.045). However, although propionic acid concentrations tended to be higher in all experimental groups than in control Group 7 without probiotic supplementation, the difference was not significant (P > 0.05).

**Table 3:** Comparison of the concentrations of four short chain fatty acids (Mean ± Standard deviation) by caecal fermented between 6 different probiotic-treating groups and an untreated group by probiotic supplementation.

SCFA	Chickens fed with a variety of probiotic supplements						Control group
	Group 1	Group 2	Group 3	Group 4	Group 5	Group 6	Group 7
Acetic acid	56.92 ± 15.46^*^	49.50 ± 6.89^*^	78.92 ± 16.41	78.17 ± 11.59	79.21 ± 3.83	93.24 ± 1.53^*^	76.60 ± 9.63
Propionic acid	31.40 ± 5.90	28.80 ± 3.01	30.47 ± 2.12	30.40 ± 2.92	29.86 ± 1.57	31.37 ± 2.88	27.92 ± 1.36
Isobutyric acid	34.77 ± 4.80^*^	28.39 ± 2.59	32.71 ± 2.11	20.32 ± 0.06	20.47 ± 0.14	24.31 ± 7.47	20.31 ± 0.90
Butyric acid	22.86 ± 3.65	20.52 ± 0.33	20.62 ± 0.15	33.73 ± 5.48	32.38 ± 2.77	43.29 ± 3.80^*^	30.09 ± 2.53

Note: the *indicate significant differences (P < 0.05) between the probiotic-treating groups and control group.

### Improvement of enzyme activity in chicken GI tract by probiotic addition

**Figure 1:**
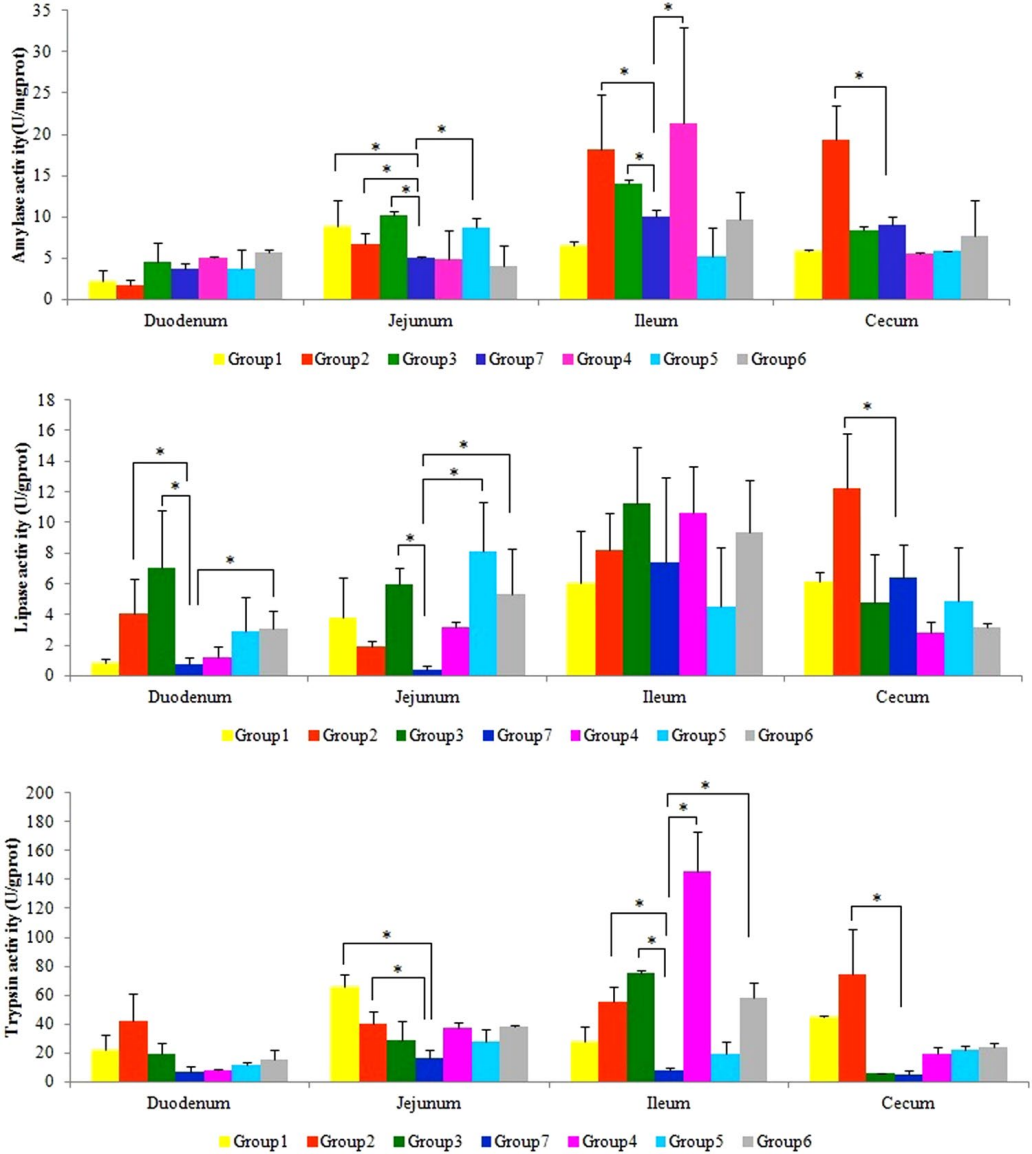
Levels of enzyme activity in chicken gut tracts. The intestinal positions are including duodenum, jejunum, ileum and cecum. (**A**) Amylase activity. (**B**) Lipase activity. (**C**) Trypsin activity. Asterisk (*) represents the significant difference of enzyme activity between certain trial groups (Group 1 to Group 6) and Group 7.

The activities of three carbohydrase enzymes, namely amylase, lipase and trypsin, are shown in Fig. 1. For the jejunum, chickens in Groups 1, 2, 3 and 5 had significantly greater amylase activity than those in control Group 7 (P = 0.016, 0.023, <0.001 and 0.028, respectively). Meanwhile, Group 3, 5 and 6 also had significantly higher lipase activity compared to control Group 7 (P < 0.001, P = 0.049 and 0.043). In addition, Group 1 and 2 had significantly higher trypsin activity compared to control Group 7 (P = 0.038 and P = 0.044). Although the trypsin activity of other experiment groups was higher than the control group, but there was no significant difference (P > 0.05). For the Ileum, the amylase activity and trypsin activity of Group 2, 3 and Group 4 were significantly higher than control Group 7 (P < 0.05), but the lipase activity of probiotic experimental groups had no significant difference compared to control Group 7 without probiotic supplemention(P > 0.05). For the cecum, the amylase activity, lipase activity and trypsin activity of Group 2 was all significantly increased compared to control Group 7 (P = 0.014, 0.019 and P = 0.023). For the duodenum, only the lipase activities in Group 2, 3 and 6 were significantly higher than control Group 7 (P = 0.036, 0.033 and 0.024), but the amylase activity and trypsin activity in all probiotic experiment groups had no significant differences compared to control Group 7 (P > 0.05).

### Sequences and taxonomic composition

The hypervariable V3 region of 16S rDNA genes was sequenced from caecum samples of individuals with probiotic treatment (n = 12) and the control group (n = 3). A total of 247,658 high-quality and classifiable reads were generated from all samples, and the number of sequences in each group varied from 34,803 to 35,957 (median = 35,379). A total of 15,074 operational taxonomic units (OTUs) were identified at the 97% sequence similarity level with high threshold identity and with an average of 2153 OTUs for each group sample. For all samples, rarefaction curves (Supplementary Figure S2) showed an increasing trend in trial groups (Group1 to Group 6) over the control group (Group 7), but the difference did not reach statistical significance. The rarefaction curves indices were highest in Group 6. This suggested that the microbial diversity of trial groups was higher than in the control group.

The Shannon and Simpson indices were applied to evaluate the diversity of gut microbiota, while the Chao1 and ACE was an indicator of species abundance. The results from the analysis of the alpha diversity metrics showed that except for Group 5, the Shannon and npShannon indices of probiotic-treated groups was increased, but Simpson index was decreased compared with control Group 7. In addition, Chao 1 and ACE displayed significantly higher index in Group 3 and Group 6 than the control Group 7 (P = 0.016, 0.023, 0.021, 0.018, respectively) (Table S3), these results suggest that probiotic additions to diet may result in an increase in the diversity of chicken intestinal bacterial flora.

**Figure 2:**
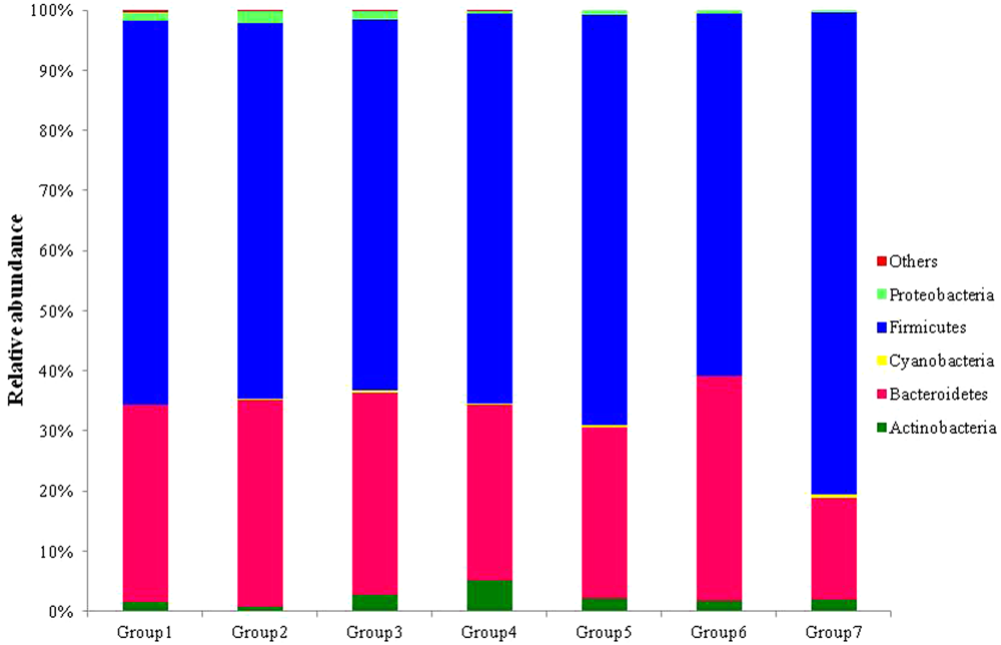
Phylum level composition of the caecal microbiome of chicken. A color-coded bar plot shows the average bacterial phylum distribution in different treatment groups and control group. The Group 1 to Group 6 refer to the probiotic-treated group, the Group 7 refer to the control group.

The overall microbial composition of the probiotic treatment group and control group differed at the phylum levels (Fig. 2), although none of these differences were statistically significant after multiple test correction. In this study, more than 99.90% of bacterial rDNA sequences were assigned to the domain Bacteria, with more than 99.00% of sequences belonging to five bacterial phyla: *Actinobacteria*, *Bacteroidetes*, *Cyanobacteria*, *Firmicutes* and *Proteobacteria*. Similarly, less than 1% of sequences obtained from all the sampled chickens were assigned to the phyla *Deferribacteres*, *TM7*, *Tenericutes*, and *Verrucomicrobia*. *Firmicutes* was the most abundant phylum (60.0–80.3%) in the caecum of chicken in all groups, and its abundance decreased in the probiotic additive group compared to the control group. *Bacteroidetes* was the second most abundant phyla (17.0–34.4%) in all groups. Unlike *Firmicutes*, after the experimental diet was supplemented with probiotics, the abundance of *Bacteroidetes* increased. In addition, the presence of *Cyanobacteria* was not obvious in caecum; it was only detected in Group 2 (0.13%), Group 3 (0.26%), Group 5 (0.45%) and control group 7 (0.48%).

**Figure 3:**
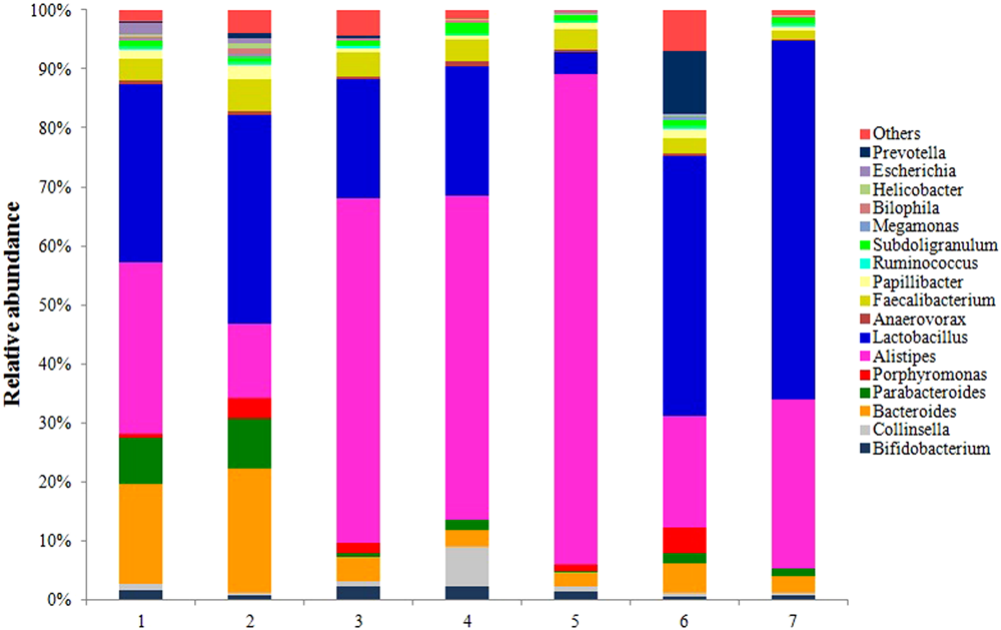
Genus-level composition of the caecal microbiome of chicken. A color-coded bar plot shows the average bacterial genus distribution in different treatment groups and control group. The number of 1, 2, 3, 4, 5 and 6 refer to the probiotic-treated group (Group 1 to Group 6), the number 7 refer to the control group (Group 7).

At the genus level, the detected sequences could be assigned to 76 different genera. The most abundant (relative abundance ≥1%) suggested the importance of bacteria (Fig. 3). In all trial groups (Group 1 to Group 6) and the control group 7, the *Alistipes* and *Lactobacillus* were the dominant genera, representing 10.45%, 4.31%, 24.91%, 23.00%, 26.20%, 5.30%, 14.48% and 10.88%, 12.26%, 8.52%, 9.28%, 1.16%, 12.48%, 30.75% of the total sequences, respectively. Meanwhile, *Bacteroides* and *Faecalibacterium* were the second most dominant genera, representing 6.14%, 7.29%, 1.80%, 1.19%, 1.21%, 1.40%, 1.47% and 1.29% and 1.84%, 1.76%, 1.62%, 1.06%, 1.23%, and 1.42% of the total sequences, respectively.

### Influence of probiotic supplements on development of chicken ceacal microbiome

**Figure 4:**
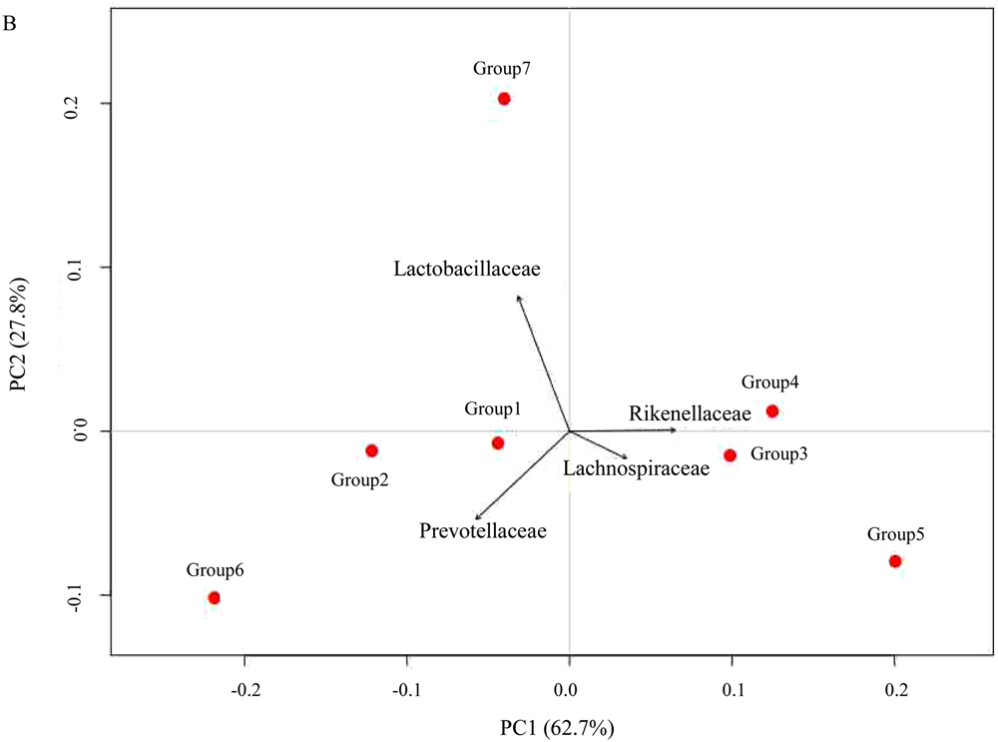
The Principal Component Analysis (PCA) of caecal samples in chickens supplemented with different probiotic additives (Group 1 to Group 6) and chicken without probiotic addition (Group 7).

Principal component analysis (PCA) highlighted a disparity between the probiotic-treated groups and the control group (Group 7) at the bacterial family level (best observed from PC1 (62.7%) and PC2 (27.8%), Fig. 4). This disparity can be attributed to *Lachnospiraceae*, *Rikenellaceae* and *Prevotellaceae* in the first direction (PC1) in *Lactobacillaceae* in the second direction (PC2). Group 1, 2, and 6 cluster together, and Groups 3, 4, and 5 cluster together; Group 7 was separated into its own cluster. Although Group 1, 2 and 6 all belong to *Prevotellaceae*, the reads number of *Prevotellaceae* were not uniformly distributed in gut libraries from the different chicken groups. Meanwhile, the sequences of members of the genus *Prevotella* were not found in Group 4 and Group 7, whereas caecal microbiota of the other five groups showed enriched sequences of the genus *Prevotella* (Fig. 3). In addition, as shown in Fig. 4, the relationship of Group 7 and the *Lactobacillaceae* family is closer. The *Lactobacillaceae* family contains the *Lactobacillus* and *Pediococcus* genus. However, the reads number of genus *Lactobacillaceae* was quite abundant in chicken caecal microbiota (on average beyond 95%), while the reads number for the genus *Pediococcus* was comparatively sparse in the gut. Only chickens from Groups 1, 2 and 6 (supplemented with *P*.*pentosaceus*) showed a low number of sequences from the genus *Pediococcus*, suggesting that *Pediococcus* may not be a common resident in chicken caecum.

As shown in Fig. 4, the relationship of Groups 3, 4, 5 to the families *Lachnospiraceae* and *Rikenellaceae* is closer. Many reads number were assigned to *Lachnospiraceae* and *Rikenellaceae* in each group; few reads number of the genera *Dorea* and *Roseburia* (*Lachnospiraceae*) were observed compared to the genus Alistipes.

### Association between bacterial phyla in chicken microbiota and SCFAs production

**Figure 5:**
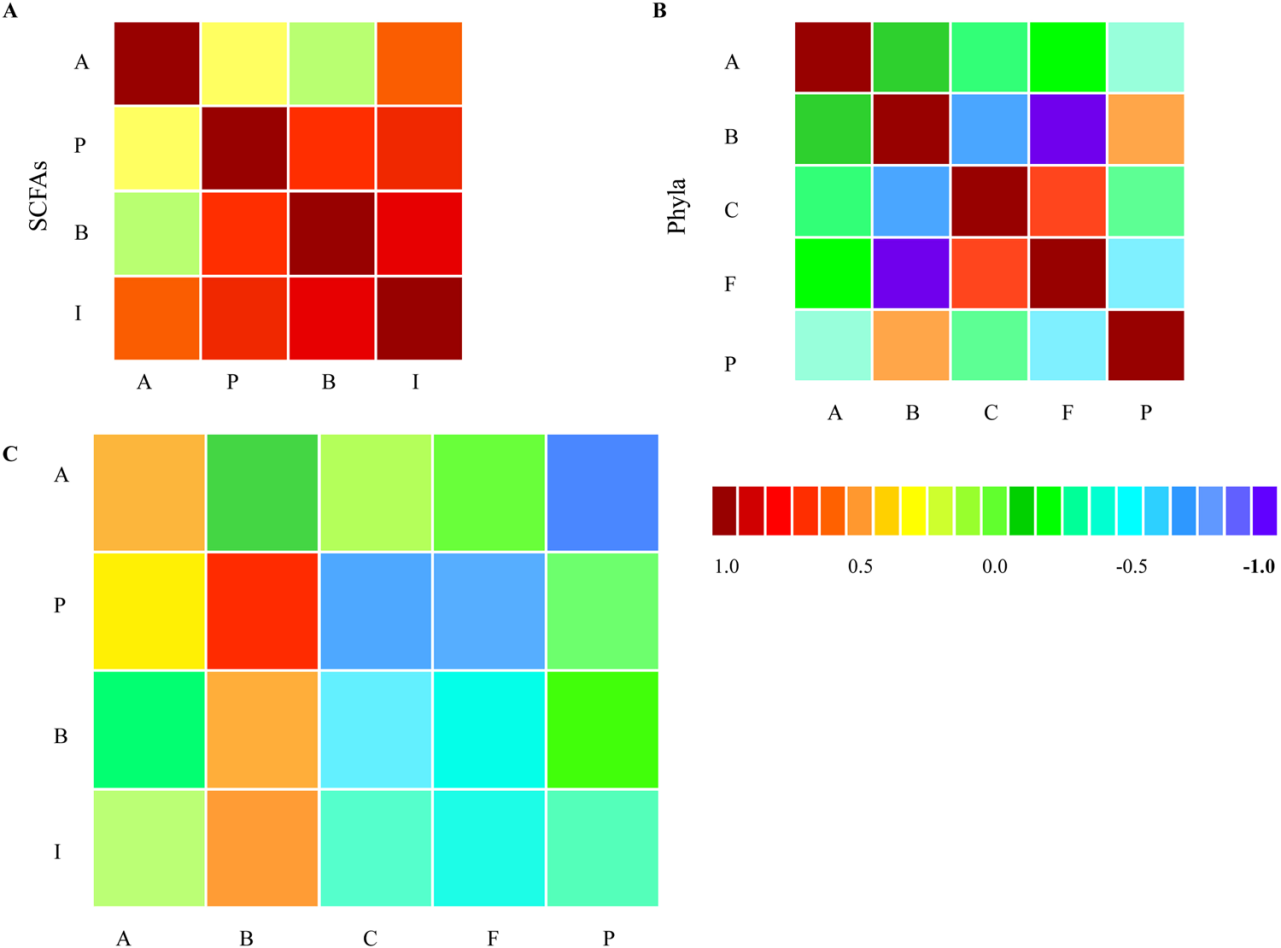
Relationships between phyla abundances and SCFA concentrations in chicken cecal samples. (**A**–**C**) Correlation matrices: (**A**) SCFAs (A, acetate; P, propionate; B, butyrate; I, isobutyrate); (**B**) Phyla (A, *Actinobacteria*; B, *Bacteroidetes*; C, *Cyanobacteria*; F, *Firmicutes*; P, *Proteobacteria*); (**C**) Cross-correlation between phylum abundances and levels of SCFAs. The color scale indicates correlation levels: Positive correlation values are in red; Negative correlation values are in purple.

Results from the Canonical Correlation Analysis (CCA) suggest that all the four types of SCFAs were positively correlated among each other (Fig. 5A). We used CCA to compare the correlation between bacterial phyla across all samples, which revealed that in sampled chickens, the phyla *Actinobacteria* and *Bacteroidetes* and *Proteobacteria* and *Bacteroidetes* were positively correlated, while the phyla *Cyanobacteria* and *Firmicutes* were positively correlated (Fig. 5B). Similarly, the sequence abundance of *Bacteroidetes* was positively correlated to the concentrations of propionate, butyrate, and isobutyrate, and increases in acetate were positively correlated to the sequence abundances assigned to *Firmicutes* (Fig. 5C). Actinobacterial sequences from all samples were positively correlated to the contents of acetate, propionate, and butyrate, while the proteobacterial sequence abundances were only positively correlated with propionate (Fig. 5C). Except for butyrate levels in Group 2, the overall SCFA production from the caecal microbiota of chickens fed with probiotic additives was higher than in chicken caeca without any probiotic addition (Fig. 1).

## Discussion

The effect of probiotics on QJM chicken growth performance was evaluated in this study. It was clear that the dietary supplementation with probiotics resulted in higher average body weight and carcass weight compared to the control group. There was no significant effect on the growth performance of chicken, except for Group 3 (*Issatchenkia orientalis* and *Bacillus macerans*), when the probiotic was administered via feed. Similarly, there have been several studies for which no positive results were found when chickens were fed with probiotic supplements. For example, Lee *et al*.^24^ and Zhang *et al*.^25^ did not find any significant difference in chicken growth performance. However, numerous studies have shown that various probiotics promote the growth performance of chickens. For example, body weight and the daily weight gain was improved by a culture of *B*. *coagulans* ZJU0616^26^ and by a mixture of *Lactobacillus reuteri*, *Bacillus subtilis* and *Saccharomyces cerevisiae* ^27^ or a single strain of *Clostridium butyricum* ^28^. However, it was difficult to directly assess different studies using probiotics because the efficacy of a probiotic application depended on many factors, including the type of strains, administration level, concentration of probiotics, application method, overall diet, and overall farm hygiene^29^. In addition, there was no significant difference among the treatment groups (Group 1- Group 6) with different mixtures of probiotic bacteria. This indicated that the quantity of *Issatchenkia orientalis* and *Bacillus macerans* was a factor for improving chicken body weight and carcass weight.

The findings of this study showed that the use of the mixed probiotic *Pediococcus pentosaceus* and *Bacillus subtilis*, *Issatchenkia orientalis* and *Bacillus macerans*, or the single probiotic *B*. *macernas* in diet could reduce the FCR of Qingjia Ma chickens. Similarly, some researchers reported that the probiotic can improve the FCR in poultry^30^. In contrast, there was no significant difference in FCR between the probiotic fed group and the control group in accordance with the reports of Bandy and Pampori^31^. Therefore, we think positive effects may be associated with specific species of probiotics.

The aroma of chicken meat is an important attribute that greatly influences consumer acceptability. It is the result of a special assortment with specific relative quantities of a mixture of different metabolites (esters, aldehydes, alcohols, ketones and acids). Aroma properties are determined by different proportions of volatile components and the presence or absence of components. In our study, 52, 60, 54, 54, 49, 55 and 43 volatile compounds were identified in the probiotic supplement group and the control group. Meanwhile, our results suggested that Group 2 and Group 6 had higher contents of (E)-2-heptenal and (E)-2-octenal than control Group 7, which implied that the two varieties might have a pleasant meat aroma. For example, 1-octen-3-ol has the characteristic odour of mushroom and a very low odour threshold^32^, and it was present in all probiotic supplementary groups except for Group 1. In addition, hexanal is the most abundant product of lipid oxidation in meats and is often chosen as an index of the level of oxidation^33^; therefore, in our study, hexanal was detected in all experiment groups and control group. However, (E,E)-2,4-Heptadienal and (E)-2-Decenal are considered important flavour-active components and were only found in Group 2 and Group 6. We hypothesize that different probiotics can affect the composition of flavour, but the specific relationships require further research.

Some researchers have reported that the amounts and types of prebiotics entering the large bowel can influence the growth of microbial populations and the production of SCFAs (predominantly acetic, butyric and propionic acid)^34, 35^. In addition, acids are among the predominant group of volatile compounds. Due to their very strong odours and lower threshold values, the SCFAs play an important role in flavour development^36^. In this study, the levels of acetic acid, a major SCFA, were reduced in the experimental treatments of Group 1 and Group 2 compared to those in the control group. The lower acetic acid concentrations could be due to the abundance of microbial populations including *Pediococcus pentosaceus*, *Bacillus subtilis*, *P*. *pentosaceus* and *Bacillus cereus var*. *albolactis*. In addition, our investigation showed a perfect positive correlation between SCFAs concentrations and digestive enzyme activities in the chicken caecal samples (r^2^ = 1.0000, p < 0.0001), The concentration of propionic acid is associated with trypsin activity with a highest canonical correlation coefficient (0.6370). In other words, with the help of probiotic supplementation in the present study, SCFA production via caecal fermentation likely has mutual benefits in the activities of carbohydrase enzymes in the chicken caecum, similar to previous reports^37–39^.

The caecum is a complex ecosystem that includes a highly varied microbiome. In recent years, many researchers have used high-throughput sequencing to investigate the gut microbial diversity of animals. In this study, at the phylum level, *Firmicutes*, *Bacteroidetes*, *Actinobacteria* and *Proteobacteria* were identified as the dominant bacteria in both probiotic-supplemented groups and the control group. These results were similar to previous studies showing that *Firmicutes* and *Bacteroidetes* were the dominant phyla in poultry caecum and ilea samples^40–42^. However, our results differed from those by Singh *et al*., who reported that *Proteobacteria* was the dominat phyla, followed by *Firmicutes* and *Bacteroidetes*, in chicken faecal samples^43^. Interestingly, in the duck^44^ and goose^45^ caecum, the dominant phyla, from high to low, are *Bacteroidetes*, *Firmicutes* and *Proteobacteria*. Similarly, Scupham *et al*.^46^ also reported that the dominant phyla in turkey caecum, from high to low, are *Bacteroidetes*, *Firmicutes* and *Proteobacteria* using a 16S rRNA clone library approach. The microbial diversity of chicken caecum differed from duck, goose and turkeys. From an evolutionary perspective, duck and geese are both *Anseriformes* poultry and they have a closer relationship than chickens and turkeys. However, from the standpoint of life habit, chickens and ducks employ roughage utilization than geese and turkeys, and ducks and geese both live in water. Therefore, we posit that the microbial diversity of gut is influenced by multiple factors including heredity, environment and diet.

In our study, we investigated that the effect of different probiotics on population structure at the genus level. Among the different genera, *Lactobacillus*, *Alistipes*, *Bacteroides* and *Faecalibacterium* were the abundant bacteria in chicken caecum. *Lactobacillus* is an important probiotic bacterium for promoting healthy and has been studied and used in medicine and the food industry for years^47^. Our results found that it was present in all probiotics-supplemented groups and the control group, ruling out the need for gut modulation of this bacteria. In addition, the microbial diversity of chicken caecum in the probiotics-supplemented group and control group was dominated by *Bacteroides*, consistent with a previous study^41, 48^. *Bacteroides* plays an important role in the breakdown of complex polysaccharides, starch and cellulose into simpler compunds^49^. In this study, treatments with various probiotics supplemented in the diet increased the abundance of *Bacteroides*. Interestingly, consistent with the result of Shaufi *et al*.^41^, we also detected the rare genera *Alistipes* and *Faecalibacterium* in all groups; these are the main bacteria involved in producing SCFA. However, using previous methods, *Alistipes* and *Faecalibacterium* was not detected.

Here, compared to the control group, we found that the abundance of *Prevotella* tended to increase with the addition of probiotics, except for Group 4 (*B*. *cereus var*. *albolactis* + *I*. *orientalis* + *L*. *plantarum*). In the rumens of goats and bovine, *Prevotella* was the abundant bacterial genus^50^ and had an important role in the utilization of carbohydrates within the gut microbial ecosystem. This might explain why the abundance of *Prevotella* in the treatment group with added *P*. *pentosaceus*.

In conclusion, the present study showed the effect of different probiotics on the growth performance and flavour of chicken, and based on 16S rRNA gene sequencing, reported the overall composition of the microbial ecosystem in the caecum of chicken with different probiotics added to the diet. Our data revealed that *Issatchenkia orientalis* and *Bacillus macerans* can significant improve the body weight and carcass weight of the chicken. Meanwhile, many volatile compounds were identified in the probiotic supplement group, and the probiotic treatment group had significant effects on the microbial community in the caecum of chicken. Although the results of the current study provide new leads for further investigation of the interaction between probiotics additives, gut microbial diversity and the meat flavour of chicken, long-term investigations of the mechanism of probiotics additive to the gut microbes of chicken are also needed.

## Electronic supplementary material

Supplementary Information
