# Supplementary material for: Effect of probiotics on the meat flavour and gut microbiota of chicken
Source: Sci Rep. 2017 Jul 25;7:6400. doi: 10.1038/s41598-017-06677-z (PMC5527115; doi:10.1038/s41598-017-06677-z)
Supplement: Supplementary file 1 — Supplementary Information [file 41598_2017_6677_MOESM1_ESM.doc]

ONLINE SUPPLEMENTAL

**Effect of probiotic on meat flavor and gut microbiota of chicken**

**Yan Wang^1a^, Jing Sun^2a^, Nianzhen Li^1^, Hang Zhong^2^, Hengyong Xu^1^, Qing Zhu^1§^& Yiping Liu^1*^**

^1^Farm Animal Genetic Resources Exploration and Innovation Key Laboratory of Sichuan Province, Sichuan Agricultural University, Chengdu Campus, Chengdu 611130, China

^2^Chongqing Academy of Animal Science, No. 51 Changlong Avenue Rongchang County, Chongqing, 402460, China

**Correspondence Author:** Yiping Liu, Institute of Animal Genetics and Breeding, Farm Animal Genetic Resources Exploration and Innovation Key Laboratory of Sichuan Province, Sichuan Agriculture University, 211 Huiming Road, Chengdu 611130, China. [liuyp578@yahoo.com](mailto:liuyp578@yahoo.com)

Qing Zhu, Institute of Animal Genetics and Breeding, Farm Animal Genetic Resources Exploration and Innovation Key Laboratory of Sichuan Province, Sichuan Agriculture University, 211 Huiming Road, Chengdu 611130, China. [zhuqing5959@163.com](mailto:zhuqing5959@163.com)

^a^ These authors contributed equally to this work.

^*^Corresponding author;^§^Co-corresponding author

**Figure S1** Dynamical increase histogram of average body weights of Qingjiaoma chickens. For each group, the body weights of each bird were monitored once per week.


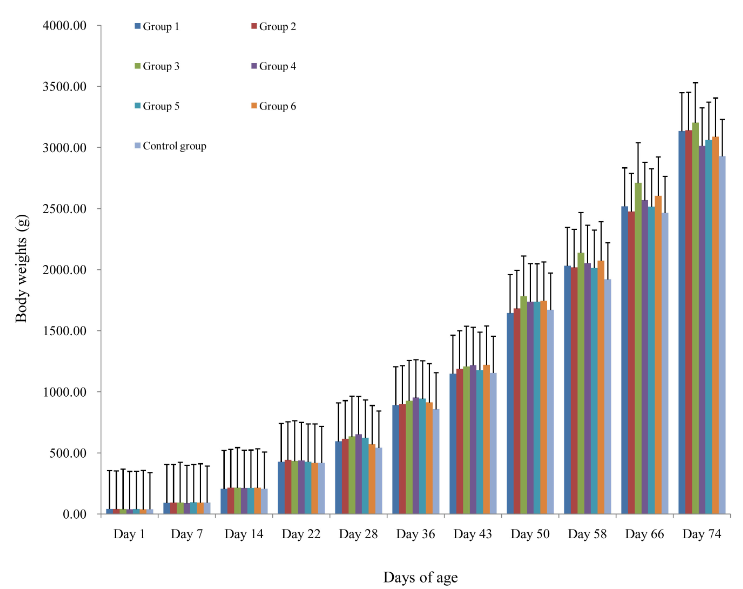


**Fig. S2.** The rarefaction curve of cecal samples in chickens supplemented with different probiotic additives (Group1 to Group6) and chicken without probiotic addition (Group7).


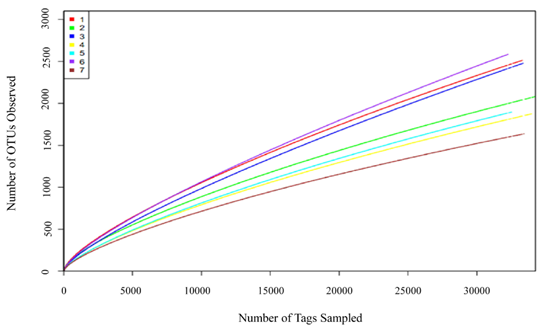


**Table S1** Top 6 bacterial strains from free-range chicken intestinal segments in specific culture medium, optimal conditions in solid-state fermentation by orthogonal design, and degree of hydrolysis to feed ingredient.

| No. | Strain | Host origin | intestinal location | Culture medium | % of strain in special culture agar | Optimal conditions of solid-state fermentation | | | | | Degree of Hydrolysis |
| --- | --- | --- | --- | --- | --- | --- | --- | --- | --- | --- | --- |
|  |  |  |  |  |  | Feed/water ratio | Inoculation amount (%) | feedstuff capacity (% ) | Fermentation temperature (°C) | Viable count (CFU/ml) |  |
| 1 | *Pediococcus pentosaceus* | Caoke | cecum | MRS Medium with 10% serum*^a^*, and enrichment by Potato-dextrose Agar*^b^* | >95% | 1:1.1 | 5 | 16 | 37 | 6.86×10^9^ | 63.41% |
| 2 | *Bacillus cereus* *var. albolactis* | Caoke | cecum | Common Nutrition Agar after enriching with 10% Serum Broth | 80% -85% | 1:1.1 | 5 | 16 | 37 | 5.41×10^8^ | 24.00% |
| 3 | *Bacillus macerans* | Qingjiaoma | cecum | Common Nutrition Agar*^c^* after enriching with 10% Serum Broth*^d^* | >80% | 1:1.1 | 5 | 16 | 42 | 6.90×10^8^ | 19.28% |
| 4 | *Lactobacillus plantarum* | Caoke | jejunum | MRS Medium*^e^* | > 90% | 1:1.1 | 10 | 16 | 37 | 8.35×10^8^ | 17.64% |
| 5 | *Bacillus subtilis* | Caoke | ileum | Common Nutrition Agar with 10% serum | > 70% | 1:1.1 | 5 | 16 | 39 | 1.60×10^9^ | 14.62% |
| 6*^f^* | *Issatchenkia orientalis* | Qingjiaoma | cecum | MRS Medium with 10% serum | 30% - 35% | 1:1.1 | 7 | 16 | 37 | 1.56×10^8^ | 23.74% |

*^a^* Bovine Serum (heat inactivated) used in this study is from New Zealand originally, Gibco®.

*^b^* The composition of Potato-dextrose Agar is 20% Peeling Boiled Potato Juice, 2% Glucose, 0.03% Penicillin/Streptomycin, 1.7% Agar without alteration of pH value.

*^c^* The composition of Common Nutrition Agar is 1% Beef extract, 2% Peptone, 1% Sodium chloride, 1.7% Bacto-Agar with pH value at 6.8;

*^d^* Serum Broth is made of 1000 ml Veal infusion agar, 10 g Peptone, 5g Sodium chloride supplemented with 100 ml Calf serum by decomplementation inactivated in water bath at 55°C, and pH value is regulated to 6.8;

*^e^* The composition of MRS Medium is 1% Casein peptone, 1% Beef extract, 0.5% Yeast extract, 2% Glucose, 0.5% Sodium acetate 3H_2_O, 0.2% Triammonium citrate, 0.1% Tween 80, 0.2% Dipotassium phosphate, 0.02% Magnesium sulfate 7H_2_O, 0.005% Manganese sulfate·7H_2_O, 1.7% Agar, final pH 5.8;

*^f^* The high degree of hydrolysis and viable count (more than ten million) made the strain TS3 the sixth candidate for use in the present study, although it had a relatively lower proportion in the original culture medium.

**Table S2** The total nutrition contents in basal feed and immunization schedules given to 420 QJM A-strain chickens.

| Ingredients (%) | 0-6w old | 7-17w old |
| --- | --- | --- |
| Corn | 58.08 | 66.58 |
| Wheat Bran | 4.21 | 0.00 |
| Puffed soybean | 0 .00 | 0.00 |
| Soy bean pulp | 26.21 | 19.23 |
| Rapeseed extraction | 2.79 | 5.26 |
| Dicalcium phosphate | 1.88 | 1.59 |
| Calcium carbonate | 0.91 | 0.83 |
| DL-Methionine | 0.17 | 0.14 |
| Mineral-vitamin premix*^a^* | 0.56 | 0.56 |
| Choline | 0.11 | 0.11 |
| Salt | 0.42 | 0.42 |
| Bentonite | 0.32 | 0.42 |
| Mildew preventive | 0.11 | 0.11 |
| Immunization Schedules*^b^* | | |
| Age of birds | Name of Vaccine | Route |
| 1 day | Marek’s vaccine (in Hatchery) | Intramuscular |
| 7 to 9 days | La Sota Newcastle vaccine and Infectious Bronchitis (1^st^ dose) | Intranasal drop + eye drops |
| 13 days | Infectious Bursal Disease Virus (IBDV) vaccine | Drinking water |
| 17 days | Bird Flu vaccine (0.3 ml per bird) | Intramuscular |
| 20 days | Flow pox (1^st^ dose) | Wing web |
| 21 days | La Sota Newcastle + Infectious Bronchitis H120 vaccines | Drinking water |
| 45 days | La Sota Newcastle + Infectious Bronchitis H120 vaccines; Newcastel disease oil-inactivated vaccine (0.3 ml per bird) | Drinking water + Intramuscular |
| 75 days | La Sota Newcastle + Infectious Bronchitis H120 vaccines | Drinking water |
| 85 days | Avian Influenz vaccine | Intramuscular |
| 120 days | La Sota Newcastle + Infectious Bronchitis H120 vaccines | Drinking water |

*^a^* Mineral-vitamin premix provided the following per kilogram of diet: vitamin A, 5,000,000 IU; vitamin D3, 200,000 IU; vitamin E, 5,000 mg; vitamin B1, 2,000 mg; vitamin B2, 2,500 mg; vitamin B6, 1,000 mg; vitamin B12, 10 mg; vitamin PP, 10,000 mg; D-Pantothenic acid, 8,000 mg; vitamin C, 25,000 mg; vitamin K, 1,500 mg; Inositol, 8,000 mg; Fe, 513 mg; Cu, 101.1 mg; Mn, 317.8 mg; Zn, 561.4 mg; I, 12.6 mg; Se, 36 mg.

**Table S3** Basic information of 16S rDNA and subsequent number of operational taxonomic units (OTUs) and richness estimates (Chao1 and ACE) at 3% distance within cecal content samples of chickens from six treatment groups (Groups 1 to 6) and the control group (Group7).

| Group ID | Clean reads | Clean data (bp) | Unique tags number | OTUs | Chao1 | ACE | Shannon | npShannon | Simpson |
| --- | --- | --- | --- | --- | --- | --- | --- | --- | --- |
| 1 | 35,382 | 10,614,600 | 7,634 | 2,521 | 8,580 | 15,784 | 4.353 | 4.544 | 0.059 |
| 2 | 35,940 | 10,782,000 | 6,947 | 2,082 | 5,808 | 10,453 | 4.014 | 4.178 | 0.081 |
| 3 | 35,462 | 10,638,000 | 7,694 | 2,478 | 10,532 | 21,210 | 3.958 | 4.175 | 0.078 |
| 4 | 35,957 | 10,787,100 | 7,497 | 1,875 | 5,460 | 9,703 | 3.626 | 3.790 | 0.095 |
| 5 | 34,895 | 10,468,500 | 7,294 | 1,896 | 5,440 | 9,773 | 3.059 | 3.258 | 0.203 |
| 6 | 34,803 | 10,440,900 | 7,586 | 2,585 | 10,616 | 24,567 | 3.981 | 4.212 | 0.086 |
| 7 | 35,219 | 10,565,700 | 5,538 | 1,637 | 4,527 | 7,888 | 3.155 | 3.312 | 0.147 |

Information for the 16S rDNA sequences was obtained using an Illumina high-throughput sequencing platform.
